# Editorial: Free radicals and antioxidants in diseases associated with immune dysfunction, inflammatory process, and aberrant metabolism

**DOI:** 10.3389/fendo.2024.1363854

**Published:** 2024-01-16

**Authors:** Hongmei Yang, Jiapeng Leng, Ning Liu, Lei Huang

**Affiliations:** ^1^The Public Experimental Center, Changchun University of Chinese Medicine, Changchun, China; ^2^Comprehensive Exposure Research Center, School of Pharmacy, Liaoning University of Traditional Chinese Medicine, Dalian, China; ^3^Central Laboratory, The Second Hospital of Jilin University, Changchun, China; ^4^ Department of Molecular Cell and Cancer Biology, University of Massachusetts Medical School, Worcester, MA, United States

**Keywords:** free radicals, oxidative stress, endoplasmic reticulum stress, immune dysfunction, metabolic disorder, inflammatory process

Free radicals, primarily composed of reactive oxygen species (ROS) and reactive nitrogen species (RNS), can originate within the body through normal cell metabolism or external factors like medication and radiation. These free radicals serve a dual role in living organisms, acting as toxic and protective agents. Free radicals play critical roles as signaling molecules at lower to moderate concentrations. However, when antioxidant defenses are compromised, excessive free radicals can lead to oxidative stress (OS) or endoplasmic reticulum (ER) stress. Oxidative stress primarily stems from impaired mitochondria and phagocytes, two major sources of free radicals and oxidants, ultimately causing damage to biomolecules such as DNA, proteins, and lipids. Recent research findings unequivocally link the overproduction of free radicals to immune and metabolic diseases, including type 2 diabetes, neurodegenerative diseases, and cancer. Patients with these conditions exhibit elevated levels of mitochondrial ROS, reinforcing this connection. Consequently, significant efforts have been directed toward developing effective interventions to mitigate these free radical-induced reactions. These interventions encompass dietary restrictions and antioxidant therapies to reduce oxidative stress and alleviate the associated disease states.

This Research Topic was designed to gather papers centered on the involvement of immune, inflammatory, and metabolic dysfunction in human diseases, focusing on the role of free radicals and antioxidants in these dysfunctions. Additionally, the topic highlighted innovative therapeutic approaches for scavenging free radicals and mitigating their detrimental effects on immune, inflammatory, and metabolic diseases. Six articles were collected to deepen our understanding of how excessive free radicals impact selected diseases and the strategies for their prevention and treatment in cellular and animal models.

Notably, the free radical nitric oxide (NO•) accumulation can trigger abnormal protein S-nitrosylation, a critical factor in the pathogenesis of diseases like neurodegenerative conditions and cancer. While significant progress has been made in understanding neurodegenerative diseases, the role of protein S-nitrosylation in cancer remains relatively unexplored, prompting further research in this area. In a study by Liang et al., protein S-nitrosylation in colorectal cancer (CRC) was investigated using biotin switch technology. They identified 19 commonly S-nitrosylated proteins associated with tumor endocrine, metabolic pathways, and apoptotic signaling pathways in CRC, suggesting that altered protein S-nitrosylation may play a role in CRC pathogenesis.

Additionally, due to the well-established link between diabetes mellitus and periodontitis, Zhao et al. conducted an in-depth review exploring the relationship between these two conditions. They examined clinical data from the perspectives of inflammatory/host immune response and oxidative stress (OS). Their analysis revealed that diabetes contributes to periodontitis susceptibility through various mechanisms, including microbiome factors, enhanced inflammatory responses, host immune factors, OS, alveolar bone resorption damage, and epigenetic changes. Furthermore, they highlighted how antioxidants can improve periodontitis prognosis by reducing OS, providing insights into the interconnected nature of endocrine, metabolic, and inflammatory diseases.

Acupuncture, a well-known alternative therapy for various ailments, was explored as a treatment approach for diseases related to diabetes mellitus in two articles within this Research Topic. Li et al. suggested that electroacupuncture could alleviate diabetic cognitive impairment by reducing hippocampal ER stress in db/db mice. Consistent with this finding, Ding et al. demonstrated that electroacupuncture improved abnormal energy metabolism in obese Zucker Diabetic Fatty rats by reducing oxidative stress. These results have clinical and social significance, offering potential early intervention and treatment options to reduce the incidence of cognitive impairment and metabolic disorders in diabetes. Furthermore, this Research Topic sheds light on other innovative therapeutic strategies for combating free radicals and their detrimental effects on immune, inflammatory, and metabolic diseases. Sidiropoulou et al. focused on the nuclear factor erythroid-derived 2-related factor 2 (NRF2), a key transcription factor regulating cellular antioxidant responses and implicated in Alzheimer’s disease (AD). They discussed the potential of natural antioxidants to activate NRF2 as a therapeutic option in AD, emphasizing the substantial antioxidant properties of these compounds. In another original research article by Liu et al., dexamethasone-loaded nanohydroxyapatite microspheres were found to exert anti-inflammatory effects by inhibiting ROS generation, IL-6, TNF-a, and IL-1b in a rat model of dental pulp injury.

In conclusion, these studies underscore the critical roles of free radicals in diseases characterized by immune dysfunction, inflammatory processes, and aberrant metabolism, focusing on elucidating the underlying mechanisms. Various avenues for antioxidant interventions have been explored and discussed by the authors. However, further research is needed to provide more data and detailed mechanisms, ultimately leading to the development of novel intervention and treatment approaches to combat these diseases.

## Author contributions

HY: Writing – original draft, Writing – review & editing. JL: Writing – original draft. NL: Writing – review & editing. LH: Writing – original draft, Writing – review & editing.

